# Metabolic characteristics and diagnostic biomarkers of malnutrition in maintenance hemodialysis patients based on WGCNA analysis

**DOI:** 10.3389/fphys.2026.1822660

**Published:** 2026-10-13

**Authors:** Bin Shi, Xuyan Wang, Jingya Zhang, Liang Du, Hongyan Luo, Haixin Xu, Jing Xie, Jia Cao, Li Cao

**Affiliations:** 1 Department of Emergency, General Hospital of Ningxia Medical University, Yinchuan, China; 2 Department of Nephrology, People’s Hospital of Ningxia Hui Autonomous Region, Yinchuan, China; 3 First Clinical Medical College, Ningxia Medical University, Yinchuan, China; 4 Department of Medical Sciences Research Institute, General Hospital of Ningxia Medical University, Yinchuan, China

**Keywords:** diagnostic markers, maintenance hemodialysis, malnutrition, metabolomics, WGCNA

## Abstract

**Objective:**

Malnutrition is a common and serious complication in maintenance hemodialysis (MHD) patients, significantly impacting their prognosis. However, the metabolic characteristics and diagnostic biomarkers of the nutritional status of MHD patients remain inadequately explored.

**Methods:**

We compared the clinical baseline characteristics between 100 MHD patients and 60 healthy controls. According to the Modified Quantitative Subjective Global Assessment (MQSGA) scores combined with clinical biochemical indicators, the MHD patients were divided into three groups: the well nourished group (Group A), the mild to moderate malnutrition group (Group B), and the severe malnutrition group (Group C). We then analyzed the differences in MQSGA scores and clinical characteristics among these three groups. Untargeted plasma metabolomics profiles were analyzed to identify differential metabolites and dysregulated metabolic pathways. Additionally, weighted gene co-expression network analysis (WGCNA) was employed in this study to screen key metabolic modules and potential biomarkers associated with malnutrition in MHD patients. Finally, the sensitivity and specificity of the core metabolites were evaluated using receiver operating characteristic (ROC) curves, and the reliability of candidate metabolite results was validated by colorimetric methods.

**Results:**

Clinical data results showed that compared with normal population, the MHD patient group exhibited decreased expression of nutritional indicators (BMI, Alb, PA, Hb) and bone metabolism-related indicators (Ca, VitD, Klotho). Furthermore, as the severity of malnutrition increased, the levels of these indicators gradually decreased. Conversely, the levels of Scr, BUN, P, CRP, iPTH, and FGF-23 gradually increased. Metabolomics results identified multiple differential metabolites among the three groups, and KEGG analysis revealed enrichment in steroid biosynthesis and the arginine-proline pathway. In addition, revealed significant alterations in both steroid biosynthesis and arginine-proline metabolism modules, characterized by markedly reduced levels of 25(OH)D_3_, Calcidiol, and Pregnanediol. Conversely, the concentrations of L-glutamate and trans-4-hydroxy-L-proline were significantly elevated (*P* < 0.05). Moreover, the results of ROC curve analysis combined with validation tests indicate that the above five metabolites may have good diagnostic performance for MHD patients with malnutrition (*P* < 0.05).

**Conclusion:**

Our findings highlight the metabolic derangements underlying malnutrition in MHD patients and propose novel biomarkers for early diagnosis. These insights may facilitate targeted interventions to improve clinical outcomes.

## Introduction

Chronic kidney disease (CKD) is a category of diseases that pose a serious threat to global public health, with most patients eventually progressing to end-stage renal disease (ESRD) (Duarte et al., 2024). Maintenance hemodialysis (MHD) serves as the core renal replacement therapy for ESRD patients, effectively extending their survival. However, long-term hemodialysis is often associated with a high prevalence of malnutrition, which is a common and serious complication (Macedo et al., 2021; Torra, 2024). Sarcopenia and protein-energy waste (PEW) remain a persistent challenge to malnutrition, affecting up to 50% of MHD patients (MHDs), but they are still not fully recognized in clinical practice (Flythe and Watnick, 2024; Zicarelli et al., 2025). Severe malnutrition in this patient population not only impairs quality of life, but also markedly elevates the risks of chronic inflammation, bone metabolism disorders, cardiovascular events and other adverse outcomes (Oliveira et al., 2019). Therefore, early detection and accurate assessment of nutritional status are crucial for improving patient prognosis.

Currently, the clinical assessment of malnutrition in MHDs is primarily based on the body mass index (BMI), serum albumin (Alb), serum nutritional parameters, and subjective scoring scales such as the MQSGA (Sivagnanam et al., 2023; Guo et al., 2024). These conventional evaluation methods are convenient for clinical application, yet they are susceptible to interference from factors such as inflammatory status, individual physical differences, and dialysis adequacy. Moreover, they fail to systematically reveal the underlying molecular metabolic mechanisms driving malnutrition progression, and lack sensitive molecular biomarkers for auxiliary diagnosis.

Metabolomics can systematically analyze small molecule metabolites in organisms and reveal the metabolic characteristics of disease progression. Accumulating evidence has confirmed that metabolic disturbance is closely implicated in the occurrence and development of malnutrition among MHDs (Ragi et al., 2023). Abnormal circulating metabolites can reflect subtle physiological and pathological changes *in vivo* earlier than routine clinical indicators (Wishart, 2019). Weighted gene co-expression network analysis (WGCNA), as an efficient systematic bioinformatics strategy, can cluster genes or metabolites into distinct co-expression modules, precisely excavate core metabolic modules closely linked to disease phenotypes (Ding et al., 2019). By applying WGCNA to metabolomic data, hub biomarkers associated with disease severity were identified, overcoming the limitations of single-omics data analysis (Zhao et al., 2025).

In this study, we integrated clinical baseline data and untargeted plasma metabolomics profiling of MHDs with different malnutrition degrees. By virtue of WGCNA, we aimed to construct malnutrition-related metabolic co-expression networks, and identify key metabolic pathways and core differential metabolites closely associated with malnutrition progression in MHD. Furthermore, the diagnostic efficacy of candidate biomarkers was evaluated through ROC curve analysis and validation experiments. The findings may provide a foundation for the early detection and targeted monitoring of the effect of nutritional intervention in this high-risk population.

## Materials and methods

### Study design

A total of 160 patients from the People’s Hospital of Ningxia Hui Autonomous Region from January 2023 to December 2025 were selected as the research subjects, including 100 MHDs from the dialysis unit as the observation group, and 60 healthy individuals undergoing routine physical examinations during the same period as the control group.

Inclusion criteria: All subjects had complete medical records meeting the follow-up requirements, with ages above 18 years; The MHDs had received regular dialysis treatment for more than 3 months, with each dialysis session lasting over 4 h.

Exclusion criteria: Patients with acute kidney injury, peritoneal dialysis, or kidney transplantation; Patients with malignant tumors, immune system disorders, diabetes, hypertension, or long-term use of glucocorticoids; Individuals who consume large amounts of vitamin D, iron supplements and protein through their diet and those suffering from severe infectious diseases.

### Sample size power calculation

The sample size was determined based on *a priori* statistical power analysis for one-way analysis of variance (ANOVA) with three independent groups. The statistical parameters were set as follows: a two-tailed significance level (α) of 0.05, a statistical power (1–*β*) of 0.80, and effect size of 0.70. The calculation results indicated that a minimum of 68 participants was needed. Considering an anticipated attrition rate of 15%, the sample size was rounded up, resulting in a final enrollment target of 80 participants. Our final cohort included 100 cases and 60 controls, providing adequate statistical power for the comparative analyses.

### MQSGA score and nutritional status grouping

The nutritional status of all MHDs was scored using the MQSGA scoring scale. This tool mainly includes seven items: dietary intake status, body mass, gastrointestinal symptoms, physiological function, sebum, muscle consumption and dialysis complications. Each indicator is strictly assigned 1–5 points based on severity, and the final score is cumulative, with a maximum score of 35 points. 7–10 are divided into the well-nourished group (Group A); 11–20 are divided into mild to moderate malnutrition group (Group B); 21–35 were divided into severe malnutrition group (Group C).

### Sample collection and preparation

Fasting venous blood samples from all subjects were collected and allowed to clot at room temperature for 1 h. Following coagulation, the samples were centrifuged at 2500 g for 4 min to separate the supernatant, which was then subjected to a second centrifugation for 10 min. The resulting clarified supernatant was aliquoted and stored at -80°C for subsequent metabolite extraction and metabolomic analysis. For metabolite extraction, plasma samples were mixed with an equal volume of methanol/acetonitrile internal standard extraction solution. The mixture was vortexed for 2 min, followed by low-temperature ultrasonication for 10 min and incubation at -20°C for 1 h. After incubation, the samples were centrifuged at 12,000 rpm for 15 min. The supernatant was collected, dried using a vacuum concentrator, and reconstituted in 160 μL of extraction solvent. The reconstituted samples were sonicated in an ice-water bath for 10 min and centrifuged again. The final supernatant was collected for liquid chromatography-tandem mass spectrometry (LC-MS/MS) analysis (15 samples per group).

### Untargeted metabolomics

The target compounds were chromatographically separated by ACQUITY UPLC HSS T3 liquid chromatographic column using Vanquish ultra-high performance liquid chromatographic spectrometer. Liquid chromatography consists of 0.1% formic acid aqueous solution in phase A and 100% acetonitrile in phase B. Each sample was loaded using a temperature-controlled autosampler. Mass spectrometer was used for primary and secondary mass spectrometry data collection. The original data were imported into the metabolomics processing software Progenesis QI for baseline filtering, peak extraction, and alignment, resulting in a data matrix containing retention time, peak intensity, and other information. To obtain accurate qualitative and relative quantitative results, the characteristic peaks are compared with those in various databases.

MHDs were divided into three groups based on MQSGA scoring, including the well-nourished group (Group A), mild to moderate malnutrition group (Group B), and severe malnutrition group (Group C). Untargeted metabolomic profiling was performed on a randomly selected subset of 15 samples from three subgroups mentioned above for preliminary differential metabolite screening. Metabolomics data were processed using MetaX software for principal component analysis (PCA) and partial least squares discriminant analysis (PLS-DA) to filter the mixed bias caused by metabolite grouping information, and construct a relationship model between metabolite expression levels and sample categories. The values of parameters R2 and Q2 describe the fitting and preprocessing capabilities of the model. Variable ImPortance in the Projection (VIP) reflects the loading weight of each metabolite in the model and assists in labeling metabolite screening. Fold Change (FC) was calculated based on the quantitative results of metabolites in the control group and the observation group. VIP >1, P<0.05, FC≥2 or FC ≤ 0.5 were used as the criteria for screening differential metabolites. Meanwhile, the corrected p-values will be clearly reported along with the original p-values, which will ensure that our identified metabolites are statistically robust.

### WGCNA network construction and metabolite co-expression module detection

We performed WGCNA analysis on all metabolites to construct biologically relevant co-expression networks. Highly correlated metabolites were clustered into distinct modules using the WGCNA algorithm. Module eigengenes (MEs) were calculated for each cluster, and hierarchical clustering was applied to define the final modules. For visualization, distinct colors were assigned to different modules and their corresponding metabolites, with gray representing unassigned metabolites. To identify biologically meaningful modules, we assessed correlations between modules and sample groups, as well as between modules and clinical phenotypes. Trait-associated modules were further evaluated using Pearson’s correlation analysis to determine their relationships with clinical parameters.

### Identification of core metabolites in key modules

The primary objective of co-expression analysis is to identify clinically relevant modules and metabolites. Using WGCNA, we assessed module significance (MS) and MEs to determine modules strongly correlated with phenotypes of interest. Hierarchical clustering and MEs-based heatmaps were generated to visualize relationships among modules, with Pearson correlation coefficients (r) and p-values used to evaluate associations between modules and clinical indicators in malnourished MHDs. To further characterize the biological relevance of key modules, we performed functional annotation and enrichment analysis of differential metabolites using the KEGG metabolomics database. Metabolites in significantly enriched pathways were screened to assess their variation across MHD malnutrition subgroups. Finally, we identified core metabolites within the most clinically relevant modules for subsequent studies.

### ROC curves analysis

Receiver operating characteristic (ROC) curve analysis was conducted to evaluate the diagnostic performance of core metabolites from key functional modules. The horizontal axis represents specificity, which refers to the ability to correctly exclude non-malnourished patients, and high specificity (>80%) helps reduce misdiagnosis. The vertical axis represents sensitivity, which refers to the ability to correctly identify patients with malnutrition, and high sensitivity (>90%) helps reduce missed diagnoses. Using the “pROC” package in R software, we calculated the area under the curve (AUC) as the primary evaluation metric. AUC values were interpreted as follows: AUClows: Excellent diagnostic performance, 0.8form<0.9: Good diagnostic performance, 0.7form<0.8: Moderate diagnostic performance, AUC<0.7: Limited diagnostic value.

### Statistical analysis

All validated data were compiled into Microsoft Excel and analyzed using SPSS 22.0 and GraphPad Prism 8.0 software. Continuous variables with normal distribution were presented as mean ± standard deviation and compared using Student’s t-test (two groups) or one-way ANOVA (multiple groups). Non-normally distributed data were analyzed using the Mann-Whitney U test. Categorical variables were expressed as frequencies or percentages (%) and compared using the χ² test. The relationship between MQSGA scores and clinical characteristics was evaluated using Spearman’s rank correlation coefficient. The threshold for statistical significance was defined as p<0.05.

## Results

### Baseline characteristics of study participants

A total of 160 participants were recruited in the present study, including 60 healthy controls (HCs) and 100 MHDs. The baseline characteristics and clinical parameters of the two groups are summarized in **Table 1**. There were no statistically significant differences in age, gender, weight, height, LDL and TG between the MHDs and HCs groups (all P>0.05). Compared with HCs, MHDs exhibited significantly lower levels of serum Alb, Hb, PA, Ca, Klotho and VitD (all P<0.05). Conversely, MHDs showed significantly higher levels of serum Scr, BUN, P, CRP, iPTH, and FGF-23 compared with HCs (P<0.01). Overall, these baseline data indicate that although MHDs are consistent with HCs in terms of basic human measurement indicators, MHDs exhibit unique clinical phenotypes, including renal dysfunction, hypoalbuminemia, calcium phosphate metabolism disorders, and chronic inflammation.

**Table 1:** Baseline characteristics of the enrolled MHDs and HCs.

Indicators	HCs (n=60)	MHDs (n=100)	*P* value
Age (years)	45.33 ± 6.70	52.25 ± 13.43	0.114
Gender (M/F)	27/33	42/45	0.33
Weight (kg)	65.25 ± 13.58	63.30 ± 9.94	0.25
Height (cm)	165.73 ± 6.91	165.74 ± 8.71	0.96
BMI (kg/m^2^)	23.67 ± 3.73	23.00 ± 2.88	0.17
Alb (g/L)	40.28 ± 0.16	37.90 ± 0.42	0.004**
Hb (g/L)	136.37 ± 10.03	109.76 ± 19.62	<0.001**
PA(mg/L)	297.45 ± 20.58	260.87 ± 82.79	0.003
Scr (umol/L)	52.39 ± 8.01	950.85 ± 285.15	<0.001**
BUN (mmol/L)	4.55 ± 1.29	21.13 ± 4.09	<0.001**
Ca (mmol/L)	2.22 ± 0.05	2.15 ± 0.18	<0.001**
P (mmol/L)	1.16 ± 0.86	1.71 ± 0.52	<0.001**
TC (mmol/L)	3.64 ± 0.38	3.50 ± 0.54	0.03*
HDL (mmol/L)	1.07 ± 0.13	0.96 ± 0.19	<0.001**
LDL (mmol/L)	1.91 ± 0.22	1.92 ± 0.61	0.92
TG (mmol/L)	1.35 ± 0.37	1.42 ± 0.24	0.18
CRP (mg/L)	0.16 (0.10, 0.15)	6.31 (0.97, 6.63)	<0.001**
iPTH (pg/mL)	56.65 (48.13, 62.15)	415.91 (184.1, 769.1)	<0.001**
FGF-23 (pg/mL)	337.82 (298.5, 344.82)	572.11 (355.75, 743.21)	<0.001**
Klotho (ng/mL)	300.32 (275.25, 315.75)	215.99 (275.75, 316.5)	<0.001**
Vit D (ug/L)	2447.46± 228.25	1885.52 ± 520.99	<0.001**

BMI, body mass index; Alb, albumin; Hb, hemoglobin; PA, prealbumin; Scr, serum creatinine; BUN, blood urea nitrogen; Ca, calcium; P, Phosphorus; TC, total cholesterol; TG, triglycerides; HDL, high-density lipoprotein; LDL, low-density lipoprotein; CRP, C-reactive protein; iPTH, parathyroid hormone; VitD, Vitamin D; FGF-23, fibroblast growth factors 23. Compared with HCs, *P<0.05, **P<0.01.

### Clinical characteristics of patients with different nutritional status among MHD group

Based on the MQSGA score, combined with clinical biochemical indicators (Alb, PA), inflammatory marker CRP, and dialysis adequacy index Kt/V, MHDs were comprehensively assessed and stratified into three groups: including the well-nourished group (Group A), mild to moderate malnutrition group (Group B), and severe malnutrition group (Group C). The baseline and clinical characteristics of the three groups are summarized according to different nutritional status in **Table 2**. Compared with Group A, patients in Group B exhibited significantly lower Alb, PA, and Hb levels; these parameters were further reduced in Group C. In addition, both Groups B and C had lower VitD and Klotho levels but higher FGF23 levels than patients with better nutritional status. Furthermore, the CRP and MQSGA scores were significantly higher in Group B and Group C compared to Group A, with the most pronounced increases observed in Group C (all P<0.01). No obvious differences were found in dialysis age and basic disease distribution across nutritional groups (P>0.05). These findings suggest that the nutritional status of MHDs is closely associated with key clinical indicators of malnutrition and bone metabolism.

**Table 2:** Clinical characteristics of different nutritional status in MHD group.

Indicators	Group A	Group B	Group C	*P* value
Cases (%)	38 (38.00)	30 (30.00)	32 (32.00)	
Gender (M/F)	21/17	16/14	16/16	0.91
Age (years)	48.41 ± 12.44	56.96 ± 13.37	52.59 ± 12.97	0.919
Dialysis age (m)	36.68 ± 20.16	49.73 ± 30.29	43.41(13.25,65)	0.49
Kt/V	1.31 ± 0.17	1.34 ± 0.24	1.44 ± 0.32	0.15
BMI (kg/m^2^)	23.56 ± 3.06	22.72 ± 2.83	22.50 ± 2.69*#	0.53
Alb (g/L)	41.36 ± 2.68	36.85 ± 2.86*	34.78 ± 3.67*#	<0.001
Hb (g/L)	117.79 ± 17.94	112.47 ± 15.17	97.66 ± 19.49*#	<0.001
PA(mg/L)	313.79 ± 70.29	267.97 ± 69.74	204.50 ± 65.13*#	<0.001
iPTH (pg/mL)	401.59 (252.3, 806.7)	429.86 (162.8, 575.5)	425.28 (374.1, 734.2)	0.92
TC(mmol/L)	3.67 ± 0.16	3.52 ± 0.48*	3.15 ± 0.28*#	<0.001
Klotho (ng/mL)	275.35 (248.8,289.7)	211.75 ± 47.45*	149.27 (140.5, 164.8)*#	<0.001
Vit D (ug/L)	2215.01 ± 455.65	1899.78 ± 365.56*	1480.87 ± 437.85*#	<0.001
FGF23 (pg/mL)	407.3 (334.6, 383.4)	577.6 (543.9, 633.6)	762.67 (683.5, 854.6)*#	<0.001
CRP (mg/L)	2.35 (0.91, 3.27)	6.39 (0.73, 8.02)	10.8 (1.11, 13.25)*#	0.008
MQSGA score	9.07 (8.0,10.0)	19.63(16.75,21.0)*	26.93 (26.0, 27.0) *#	<0.001

Results expressed either as mean ± standard deviation or as median (Q1, Q3). *Compared with Group A, P<0.05; ^#^Compared with Group B, P<0.05.

### Correlation between nutritional status indicators and MQSGA score in MHDs

We further analyzed the correlation between the above indicators of statistical difference and the MQSGA score to better explain the covariates affecting malnutrition factors. As shown in **Table 3**, we found that MQSGA score was negatively correlated with PA, Alb, Hb, Klotho and VitD, but positively correlated with FGF23. These findings provide new perspectives for understanding the role of nutritional status in the disease progression of MHDs.

**Table 3:** Correlation between nutritional status indicators and MQSGA scores.

Variable	MQSGA score	
	R value	P value
BMI (kg/m^2^)	0.11	0.24
PA (mg/L)	-0.55	<0.001
Alb (g/L)	-0.76	<0.001
Hb (g/L)	-0.43	0.005
FGF23 (pg/mL)	0.59	<0.001
Klotho (ng/mL)	-0.55	<0.001
VitD (ug/L)	-0.60	<0.001

Pearson correlation and linear regression were used to analyze the correlation between MQSGA score and nutritional status indicators in MHDs.

### Analysis of the overall metabolomics results in MHDs

We selected 15 patients from each of three groups with different nutritional statuses in MHDs, and used the UPLC-MS platform to detect differentially expressed metabolites (DEMs) among the groups. The statistical analysis results revealed a total of 2302 DEMs after filtering RSD <30% in quality control (QCs) (**Figure 1A**), including 1060 DEMs in group A compared with group B, 1061 DEMs in group A compared with group C, and 181 DEMs in group B compared with group C (**Supplementary Figures 1A–C**). Venn analysis of DEMs across the three groups revealed evident intergroup overlap, and a total of 96 metabolites were screened for subsequent investigation (**Figure 1B**). All DEMs were annotated against the KEGG database, comprising 54 amino acid derivatives, 146 lipid derivatives, and 42 nucleotides derivatives (**Figure 1C**). To explore the enrichment characteristics of DEMs among groups, the top 20 enriched metabolic pathways were identified, mainly involving arginine and proline metabolism, purine metabolism, D-Amino acid metabolism and steroid biosynthesis (**Supplementary Figures 1D–F**).

**Figure 1:**
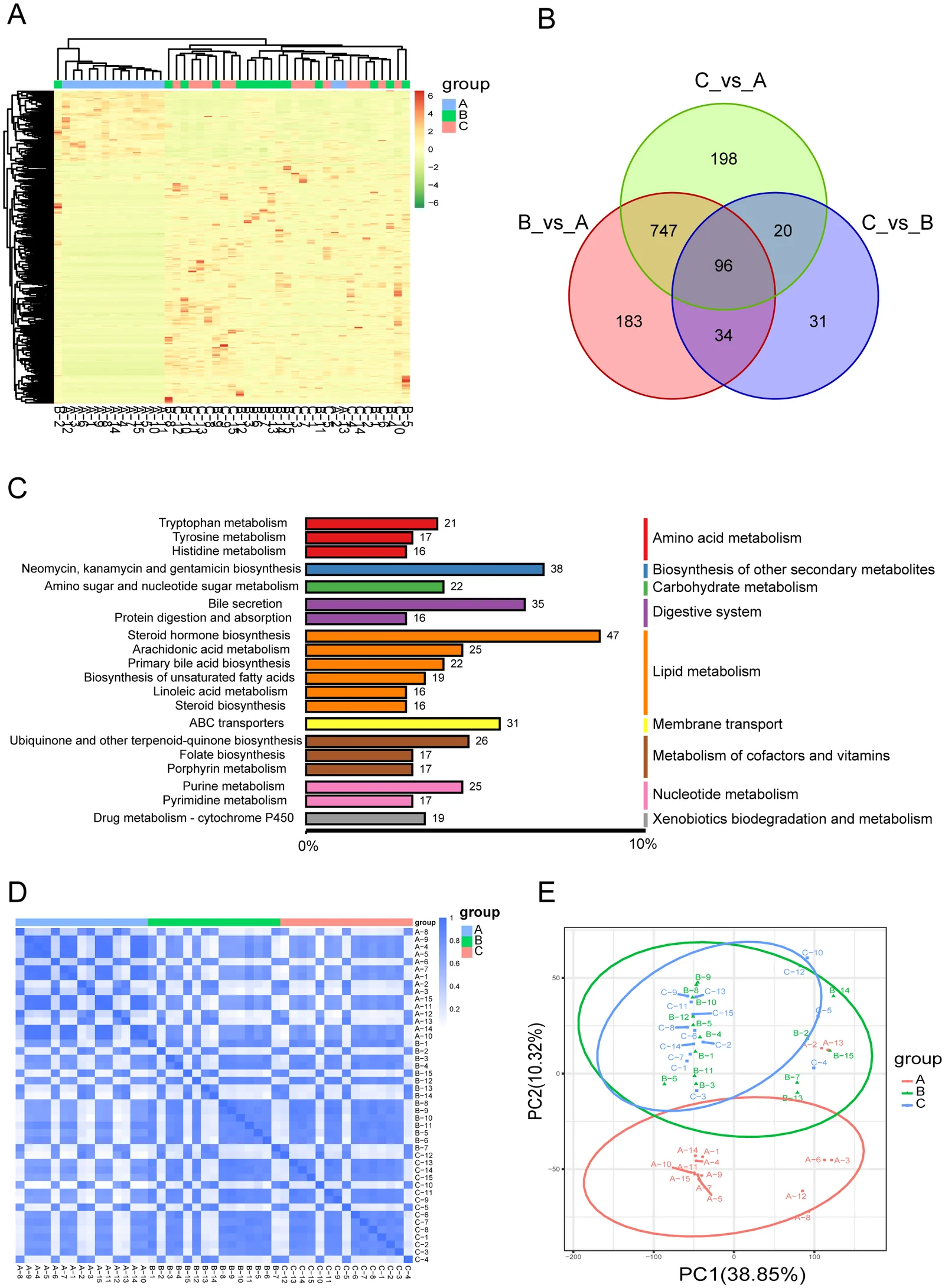
Data quality evaluation of overall metabolome. **(A)** Heatmap of DEMs, red represents the upregulated metabolites, while green represents the downregulated metabolites. **(B)** Venn diagram illustrating the overlap of DEMs across the three nutritional groups. **(C)** KEGG enrichment analysis highlights the involvement of DEMs in pathway such as amino acid derivatives, lipid metabolites derivatives and nucleotides derivatives. **(D)** Correlation heatmap demonstrating intragroup reproducibility of samples. **(E)** PCA diagram of all samples. Group A represents the well-nourished group, Group B represents the mild to moderate malnutrition group, and Group C represents the severe malnutrition group.

Spearman rank correlation analysis was applied to evaluate biological reproducibility of intragroup samples. As presented in **Figure 1D**, intragroup correlation coefficients were higher than intergroup values, demonstrating favorable sample repeatability within each group. Principal component analysis (PCA) of QC samples showed that the cumulative explanatory rate of the first two principal components was 49.17%, with PC1 accounting for 38.85% and PC2 for 10.32%. Distinct separation was observed between and within groups (**Figure 1E**). Collectively, all samples exhibited good reproducibility, and the identified differential metabolites were credible.

### Stability analysis of study cohort using OPLS-DA

OPLS-DA is a discriminant analysis method in multivariate data analysis techniques. To demonstrate the differences between the well-nourished group and the malnourished group, the discriminant analysis of metabolic data among the above groups was completed by establishing the OPLS-DA model. After seven rounds of cross-validations, the results revealed distinct and independent clustering between Group A and Group B or Group C. Specifically, the R²Y and Q²Y values for A vs B were 0.992 and 0.817, while those for A vs C were 0.972 and 0.815. The Q2Y values of both comparisons exceeded 0.5, indicating that the established OPLS-DA model was stable and reliable, and possesses satisfactory fitting ability and predictive capacity (**Figures 2A, B**). Permutation testing was applied to further verify model reliability. Sample group labels were randomly rearranged, and OPLS-DA models were repeatedly constructed to calculate R²Y and Q²Y values. After several iterations, valid results were acquired, as presented in **Figures 2C, D**. Therefore, these findings indicate that this model can be effectively used for subsequent differential metabolite searches and analyses.

**Figure 2:**
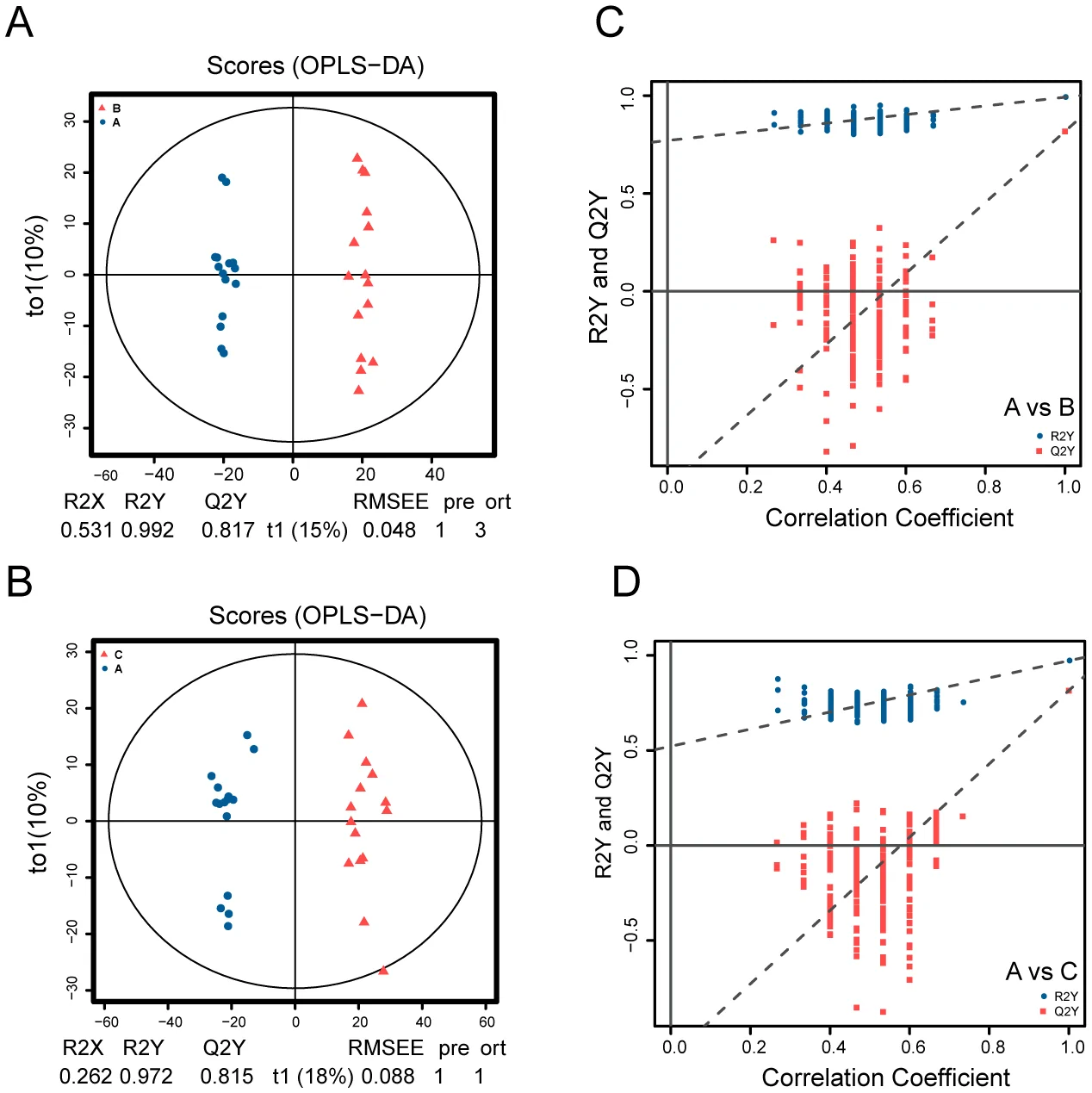
OPLS-DA score plot and permutation test. **(A, B)** OPLS-DA score chart for metabolite arrangement test of plasma samples in each group (A vs B, A vs C). **(C, D)** OPLS-DA score scatter plot of permutation test. The closer the values of R2Y and Q2Y are to 1, the more reliable and stable the model is.

### Identification of key modules based on WGCNA cluster

WGCNA is traditionally applied to gene expression profiles, and has been widely adopted for metabolomic data analysis in recent years (Zhao et al., 2024; Zhou et al., 2024). It builds weighted networks based on pairwise correlations to detect closely clustered metabolites. This approach effectively identifies metabolite modules linked to clinical phenotypes, which is difficult to accomplish via conventional univariate and multivariate analysis (Ji et al., 2024). According to the WGCNA algorithm, after appropriate normalization processing of the data, a sample tree diagram is constructed from the metabolite expression profile based on the correlation matrix and adjacency matrix (**Figure 3A**). Using predefined parameters (metabolite count ≥30, module similarity threshold=0.25), dynamic module cutting and merging were performed, yielding 19 distinct co-expression modules. Each module was assigned a unique color, while unassigned metabolites were grouped into the gray module (**Figure 3B**). To explore potential links between metabolite modules and disease mechanisms, we analyzed their correlations with clinical traits. A module-trait relationship heatmap revealed that, among the 19 modules, the yellow module exhibited the strongest negative correlation with MQSGA (r=-0.81, P = 1e-11), whereas the brown module showed the highest positive correlation (r=0.72, P = 2e-08) (**Figure 3C**). Intriguingly, feature metabolite adjacency heatmaps demonstrated a significant negative correlation between the yellow and brown modules, along with strong interactions between these modules and others (**Figure 3D**). These findings highlight the yellow and brown modules as key functional networks strongly associated with malnutrition, suggesting their potential biological significance and warranting further investigation.

**Figure 3:**
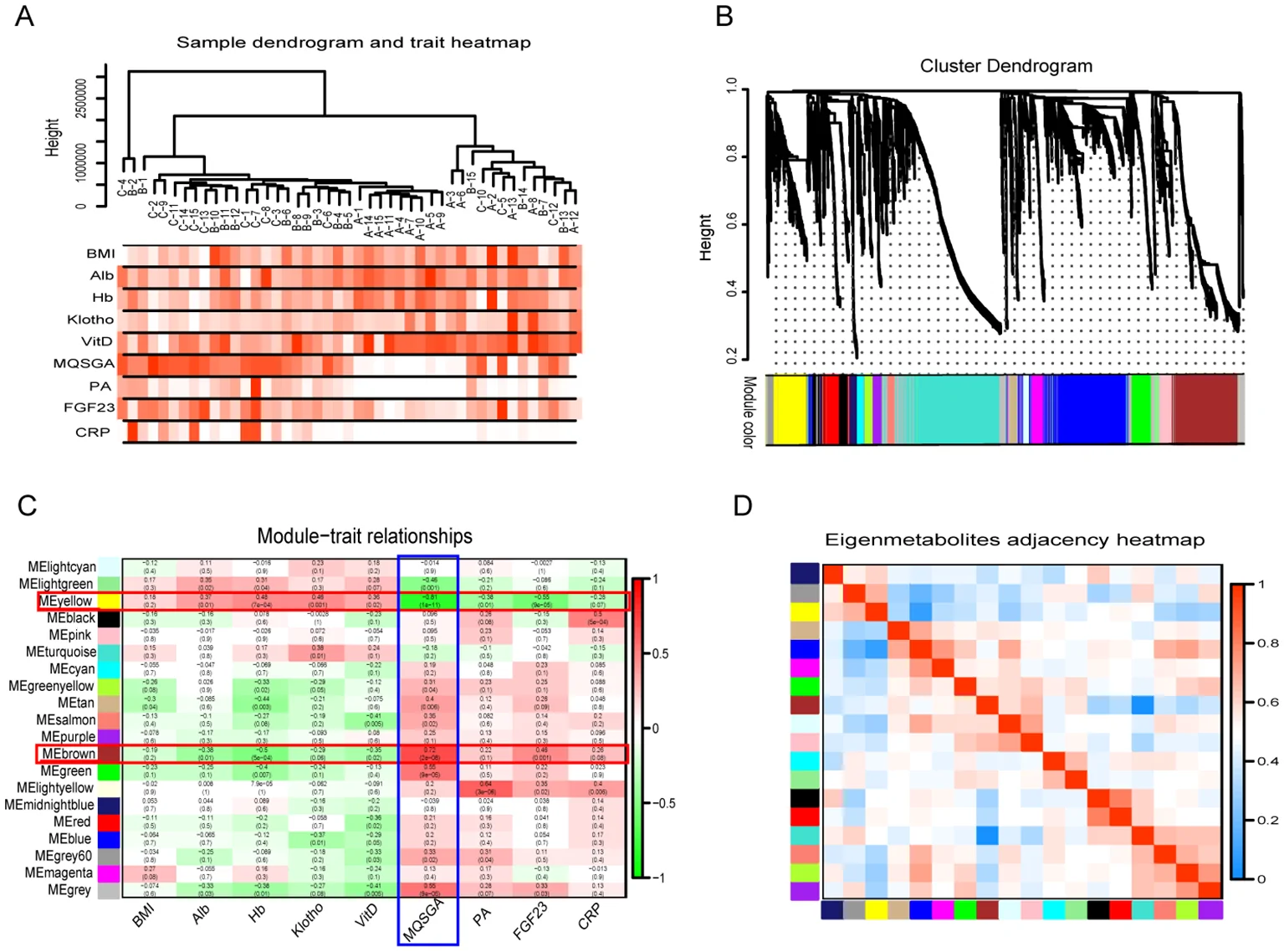
The relationship between modules and clinical traits. **(A)** Hierarchi*ca*l clustering tree of all metabolites. **(B)** The clustering dendrogram of the average network adjacency for the identification of metabolite co-expression modules. **(C)** Heatmap of module-trait relationship. Red indicates high adjacency (positive correlation) while green indicates low adjacency (negative correlation). The key yellow modules and brown modules for analysis are marked with red box lines, and the MQSGA clinical traits are marked with blue box lines. **(D)** Eigenmetabolites adjacency heatmap. Each row corresponds to a trait module, each column represents a trait, and each module contains the corresponding correlation and p-value.

### Analysis of hub metabolites of yellow modules

Considering the close correlation between the yellow module and malnutrition characteristics, we further analyzed this module and screened core metabolites. KEGG enrichment analysis classified metabolites in this module into three main groups: fatty acyls, steroids and prenol lipids (**Figure 4A**). Steroid biosynthesis, primary bile acid biosynthesis and porphyrin metabolism ranked the highest among the top 20 enriched pathways (**Figure 4B**). To visualize metabolite abundance patterns, we generated a heatmap with rows and columns representing metabolites and samples, respectively. The results demonstrated metabolite levels were generally lower in Group B and Group C than in Group A. Metabolites involved in steroid biosynthesis showed the most obvious decline, such as 25-hydroxyvitamin D_3_ (25(OH)D_3_, neg_7575), Calcidiol (neg_8032), and Pregnanediol (neg_7519) (**Figure 4C**). Network visualization is also a powerful tool for identifying core metabolites in WGCNA modules. To further elucidate the functional relevance of these metabolites, we constructed a protein-protein interaction (PPI) network based on five key signaling pathways. Metabolites involved in multiple pathways were regarded as core candidates associated with MHD-related malnutrition. Notably, 25(OH)D_3_ and Calcidiol jointly participated in steroid biosynthesis, tuberculosis pathway and parathyroid hormone synthesis, suggesting their vital regulatory functions (**Figure 4D**). To validate these findings, we performed violin plot analysis comparing the expression levels of 25(OH)D_3_, Calcidiol, and Pregnanediol across the three groups. The results confirmed the above metabolites were markedly decreased in Group B and Group C, with the lowest values detected in Group C (**Figure 4E**). These findings strongly implicate these metabolites in the metabolic dysregulation underlying MHD-associated malnutrition, warranting further investigation into their mechanistic roles.

**Figure 4:**
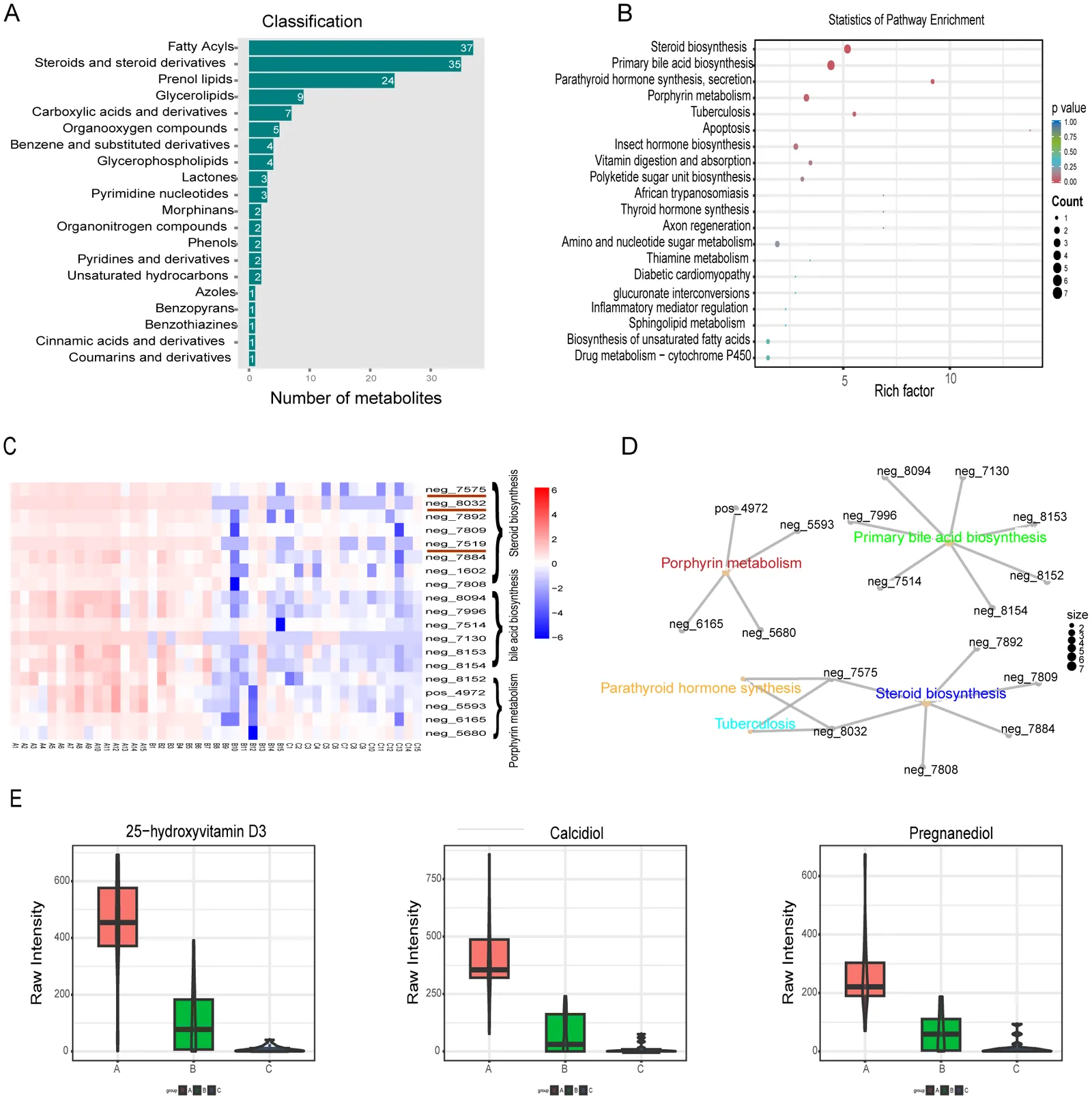
Functional analysis of core metabolites in the yellow module. **(A)** The classification bar chart of metabolites, showing significantly enriched pathways. **(B)** Top 20 enriched metabolic pathways, with bubble size representing pathway impact and color indicating significance level. **(C)** Heatmap visualization of metabolite abundance patterns. We marked the significantly altered differential metabolites in the steroid synthesis pathway with red underlines, where neg_7575 represents 25(OH)D_3_, neg_8032 represents Calcidiol, and neg_7519 represents Pregnanediol. **(D)** PPI network of key metabolites and their associated pathways. **(E)** Violin plots comparing serum levels of 25(OH)D_3_, calcidiol, and pregnanediol across nutritional status groups.

### Analysis of hub metabolites of brown modules

The brown module was also strongly correlated with malnutrition traits. Therefore, we performed KEGG classification and pathway enrichment analyses on the differential metabolites in this module. The results showed that these metabolites were mainly enriched in carboxylic acid derivatives, fatty acyls, organic oxygen compounds, and prenol lipids (**Figure 5A**). Further pathway analysis of differential metabolites (VIP>1, P<0.05) identified three significantly enriched metabolic pathways: purine metabolism, arginine and proline metabolism, and inositol phosphate metabolism (**Figure 5B**). A heatmap was generated to compare the abundance of key metabolites in these pathways across the three groups. Metabolite levels were markedly upregulated in Group B and Group C compared with Group A, with the highest upregulation observed in Group C (**Figure 5C**). Among these metabolites, L-glutamate (Glu, neg_1061) and trans-4-hydroxy-L-proline (T-4-Hyp, neg_1179), two key molecules in arginine and proline metabolism, showed the most striking alterations. To further explore their functional interactions, we also constructed a PPI network. The results indicated that L-glutamate served as a core metabolite involved in multiple metabolic pathways, including arginine and proline metabolism, butanoate metabolism, and C5-branched dibasic acid metabolism (**Figure 5D**). Finally, violin plot analysis confirmed that L-glutamate and T-4-Hyp levels were significantly elevated in Groups B and C compared to Group A, with the highest expression observed in Group C (**Figure 5E**). These findings underscore the critical role of these metabolites in the metabolic alterations associated with malnutrition, suggesting their potential as biomarkers or therapeutic targets.

**Figure 5:**
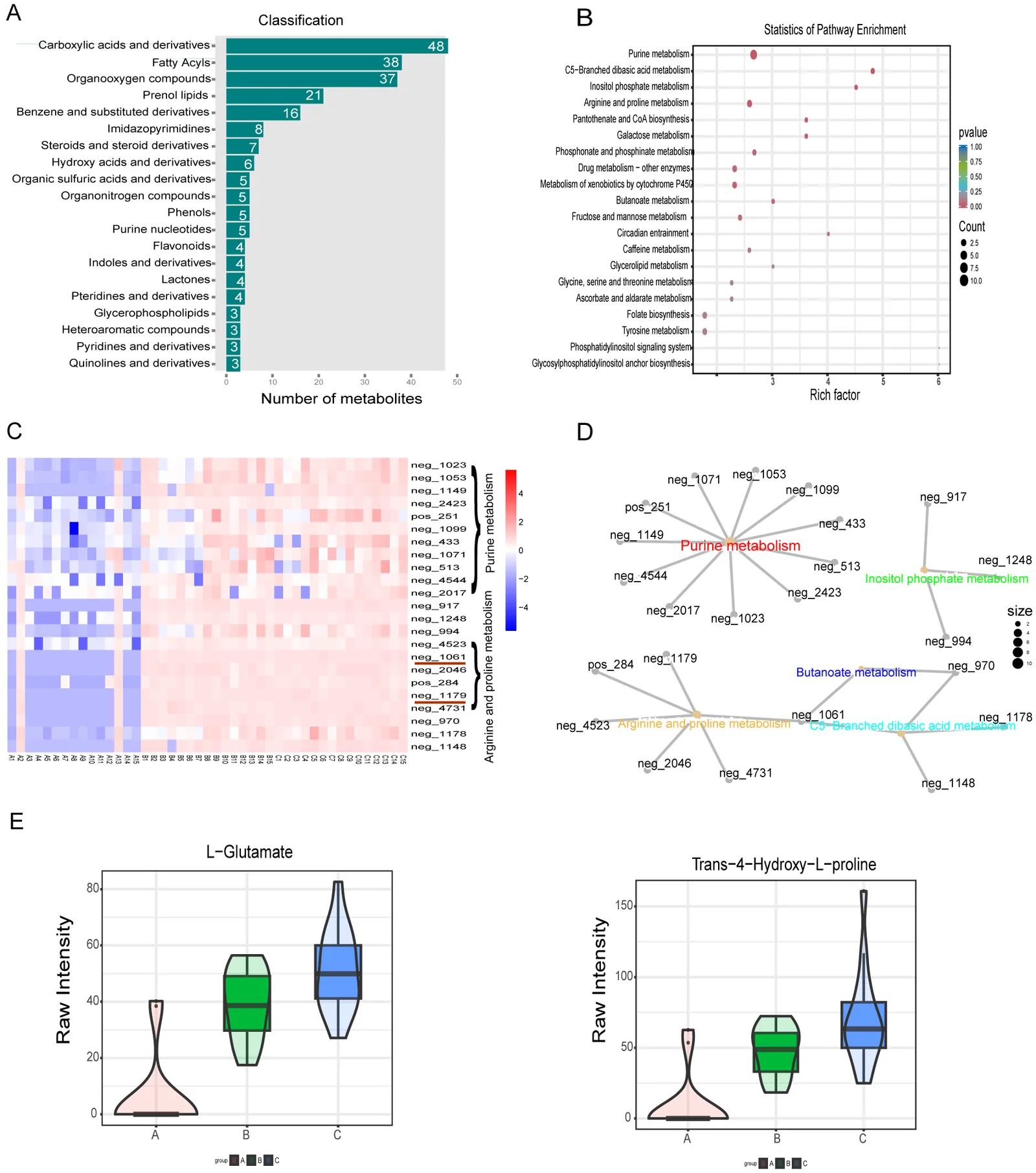
Functional characterization of core metabolites in the brown module. **(A)** KEGG pathway classification of identified metabolites. **(B)** Bubble enrichment diagram of the top 20 differential metabolic signaling pathways in the brown module. **(C)** Heatmap visualization of differentially abundant metabolites. The red underline marks the metabolites with significantly different changes in the arginine and proline pathways, among which neg_1061 represents L-glutamate, neg_1179 represents T-4-Hyp. **(D)** PPI network diagram of metabolites and pathways. **(E)** The violin diagram shows the expression levels of L-glutamate and T-4-Hyp in the three groups.

### Diagnostic performance of core metabolites

Receiver operating characteristic (ROC) curve analysis was performed to evaluate the diagnostic performance of five candidate metabolites in distinguishing between nutritional status groups. The area under the curve (AUC) was used to evaluate diagnostic performance: 25(OH)D_3_ (AUC = 0.92, 95% CI = 0.758-1); Calcidiol (AUC = 0.964, 95% CI = 0.876-1); Pregnanediol (AUC = 0.96, 95% CI = 0.876-1); L-glutamate (AUC = 0.938, 95% CI = 0.822-1); T-4-Hyp (AUC = 0.916, 95% CI = 0.762-1) (**Figure 6**). Their 95% CI all exceeded 0.75, indicating good discriminatory ability in this cohort. Notably, these ROC analyses were conducted exploratory using data from the same metabolomics discovery cohort, which may have overestimated the performance. Therefore, findings require validation in independent external cohorts to confirm their reliability. Subsequently, we measured the plasma levels of free Glutamate and Hydroxyproline in MHDs in the well-nourished group (Group A) and the severe malnourished group (Group C) using colorimetric assays. We supplemented the verification results of external data and found that plasma concentrations of Glutamate and Hydroxyproline were significantly higher in Group C compared with Group A, which was consistent with the findings of the metabolomics analysis (**Figures 7A, B**). In summary, the above results suggest that these five candidate metabolites may serve as potential biomarkers for clinical assessment of malnutrition in this patient population.

**Figure 6:**
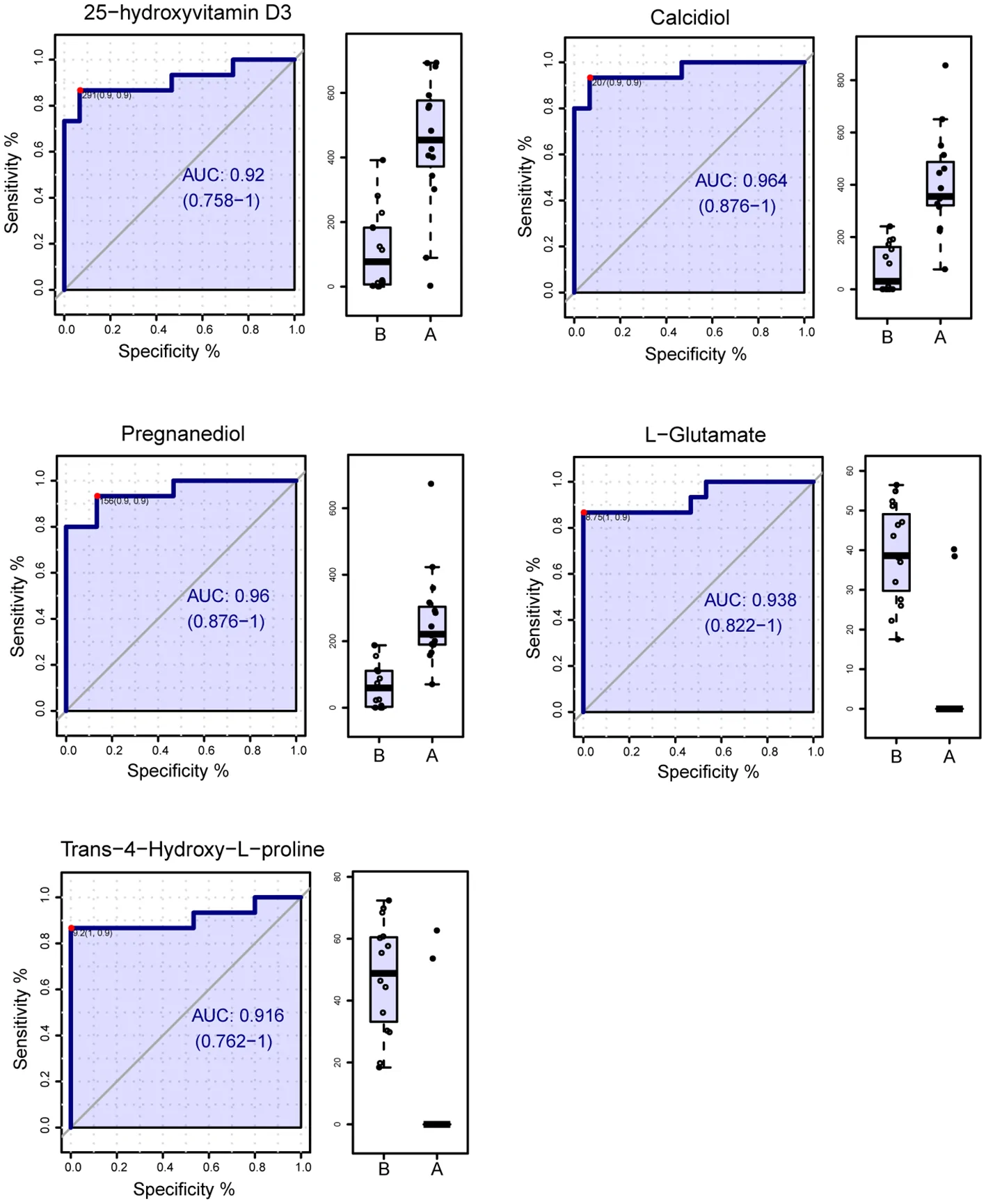
ROC curve analysis of core metabolites. The figure shows the ROC curve with a 95% confidence interval. Optimal cutoffs threshold were selected by maximizing the Youden index (AUC >0.7 considered clinically relevant). Significant metabolites had AUC >0.9 with p <0.05.

**Figure 7:**
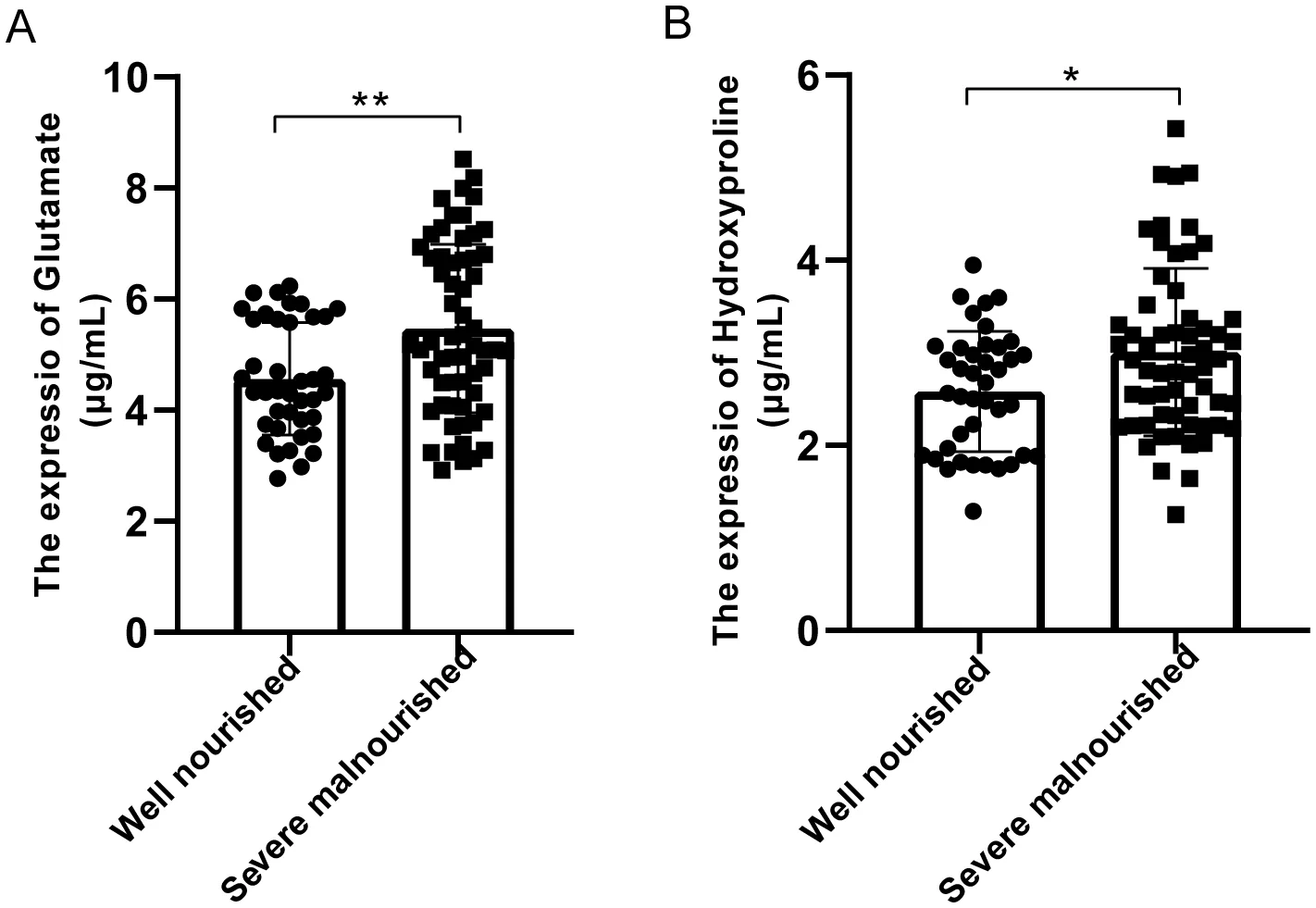
Relative expression of candidate amino acids. **(A)** The expression differences of Glutamate between the well-nourished group and the severe malnourished group were detected by colorimetry. **(B)** The expression levels of hydroxyproline in the two groups.

## Discussion

In this study, through a comprehensive analysis of multiple clinical malnutrition indicators, we found that there were significant differences between MHDs and healthy individuals. Metabolomic profiling stratified by nutritional status systematically revealed that metabolic disorders in MHDs trigger distinct alterations in pivotal metabolites and related pathways (Fonseca et al., 2023). Our results demonstrated disrupted metabolism of amino acids, lipids and steroids in malnourished MHDs. Notably, decreased levels of essential amino acids may contribute to impaired catabolic and anabolic responses in muscles (Murtas et al., 2022). Lipid metabolism disorders may exacerbate energy deficiency in patients with malnutrition (Song et al., 2025). These findings emphasize the complex interplay between metabolic dysfunction and malnutrition. However, most studies have focused on individual metabolites rather than systemic metabolic networks. WGCNA was performed to explore metabolic networks governing malnutrition, and key modules and hub metabolites closely correlated with nutritional status were screened. The most striking variations were detected in steroid biosynthesis-related 25(OH)D3 and calcidiol, as well as L-glutamate and T-4-Hyp involved in arginine and proline metabolism pathway.

This study found that plasma levels of VitD metabolites decreased significantly with worsening malnutrition in MHDs. This decline was accompanied by parallel decreases in BMI, Hb, Alb, PA and Klotho protein levels, indicating a close association between VitD metabolic disorders and nutritional status in MHDs. 25(OH)D3 and calcidiol represent sequential metabolic precursors of VitD and share similar activation pathways and signaling mechanisms (Norman, 2012; Vieth, 2020). This finding aligns with previous reports by Wenbin et al (Wenbin and Luping, 2023), who demonstrated significantly higher prevalence of VitD deficiency in malnourished MHDs compared to their well-nourished counterparts. As a fat-soluble steroid hormone, the active form of VitD (25(OH)D_3_), not only regulates calcium-phosphorus metabolism and bone homeostasis, but also plays a critical role in muscle metabolism pathways (Yamada et al., 2022). Studies have shown that VitD deficiency disrupts intracellular calcium homeostasis in skeletal muscle cells, inhibits sarcoplasmic reticulum calcium release, and impairs myofiber contractile function. These changes are directly linked to the muscle atrophy and protein-energy wasting (PEW) commonly observed in MHDs (Nassar et al., 2022; Zhou et al., 2025). Pregnanediol, another key steroid hormone identified in our study, also showed significant reduction with worsening nutritional status. This observation is consistent with existing literature demonstrating decreased progesterone secretion in malnutrition states, potentially reflecting impaired pregnanediol synthesis capacity (Kuokkanen et al., 2016). These findings collectively suggest that steroid hormone metabolism plays a crucial role in the development and progression of MHD malnutrition.

In addition, decreased Klotho protein levels were accompanied by reduced VitD metabolites in this study. As a coreceptor for FGF23, the downregulation of Klotho expression further diminishes the synthesis of 25(OH)D_3_. Previous studies have demonstrated that the VitD/Klotho/FGF23 regulatory axis triggers mineral metabolism disorders in MHD patients with ESRD (Contreras-Bolívar et al., 2021). This axis modulates inflammatory responses and accelerates skeletal muscle proteolysis, serving as one of the core mechanisms contributing to malnutrition and poor clinical prognosis (Dey et al., 2023). Therefore, we speculate that the progression of malnutrition in MHDs may be associated with metabolic abnormalities induced by VitD deficiency, chronic inflammation, and toxin accumulation, which may be mediated by specific metabolic signatures.

In contrast to the decline of VitD metabolites, plasma levels of L-glutamate and hydroxyproline were markedly elevated in malnourished MHD patients and positively correlated with malnutrition severity. Subsequent colorimetric verification was consistent with the results of metabolomics analysis, suggesting that these two metabolites participate in the progression of PEW. As the most abundant free amino acid in the human body, L-glutamate acts as an essential substrate for protein synthesis, and a precursor of glutamine and tricarboxylic acid cycle intermediates, participating in energy metabolism and oxidative stress regulation (Holeček, 2023). Research has found that glutamate metabolism is blocked in malnourished MHDs, leading to abnormal metabolism of branched-chain amino acids and insufficient energy production, which further exacerbates PEW (Wunderle et al., 2024). When MHD patients experience severe malnutrition, the body may be in a state of hypercatabolism and microinflammation, leading to increased breakdown of muscle proteins and the release of more intracellular glutamate due to cellular damage. T-4-Hyp, a hydroxylated derivative of L-proline, demonstrates significant accumulation in MHDs. This metabolic retention contributes to systemic disturbances through dual mechanisms, including activation of inflammatory pathways and negative feedback regulation of circulating L-proline, ultimately promoting protein catabolism (Chen et al., 2020; Hu et al., 2021). These findings suggest that T-4-Hyp and L-glutamate may serve as complementary biomarkers, with their combined detection of the two metabolites can improve diagnostic accuracy for evaluating nutritional status in MHDs.

This study systematically clarified the dynamic changes in routine nutritional indicators and bone metabolic indicators across different severities of malnutrition in MHDs, establishing a solid foundation for relevant clinical phenotypes. Furthermore, we innovatively combined metabolomics with WGCNA strategy to identify key metabolic modules such as steroid biosynthesis and arginine-proline metabolism linked to malnutrition in MHDs. This approach moves beyond the analysis of individual metabolites to focus on systemic metabolic networks, enabling a more comprehensive understanding of metabolic disorders underlying MHD-related malnutrition. Finally, we screened and validated plasma metabolic biomarkers with favorable diagnostic performance. The findings provide novel potential molecular targets for early warning and severity assessment of malnutrition in MHD.

Nevertheless, several limitations of this study should be acknowledged. Firstly, this is a cross-sectional study that only reveals correlations between metabolite alterations and malnutrition, without confirming causal relationships. Secondly, the research samples were from a single center. Although independent external cohorts were used for validationy, there was a lack of long-term prognostic follow-up. This might have limited the universality of the research results. Furthermore, the specific molecular mechanisms driving metabolic changes were not fully explored. In the future, large-scale multicenter cohorts are required to validate our conclusions. Further longitudinal and interventional studies are also needed to verify the improvement effects of measures interventions such as VitD supplementation and amino acid metabolism regulation on the nutritional status in MHDs.

## Data Availability

The datasets generated in this study are not publicly available due to privacy and ethical restrictions for patient‑derived serum metabolomic data. The processed data supporting the findings of this study are provided within the article and supplementary materials. Requests to access relevant datasets may be directed to the corresponding author.
