# Supplementary material for: Metabolic characteristics and diagnostic biomarkers of malnutrition in maintenance hemodialysis patients based on WGCNA analysis
Source: Front Physiol. 2026 Oct 13;17:1822660. doi: 10.3389/fphys.2026.1822660 (PMC13710070; doi:10.3389/fphys.2026.1822660)
Supplement: Supplementary file 1 [file Image1.pdf]

Supplementary Figure and Legend

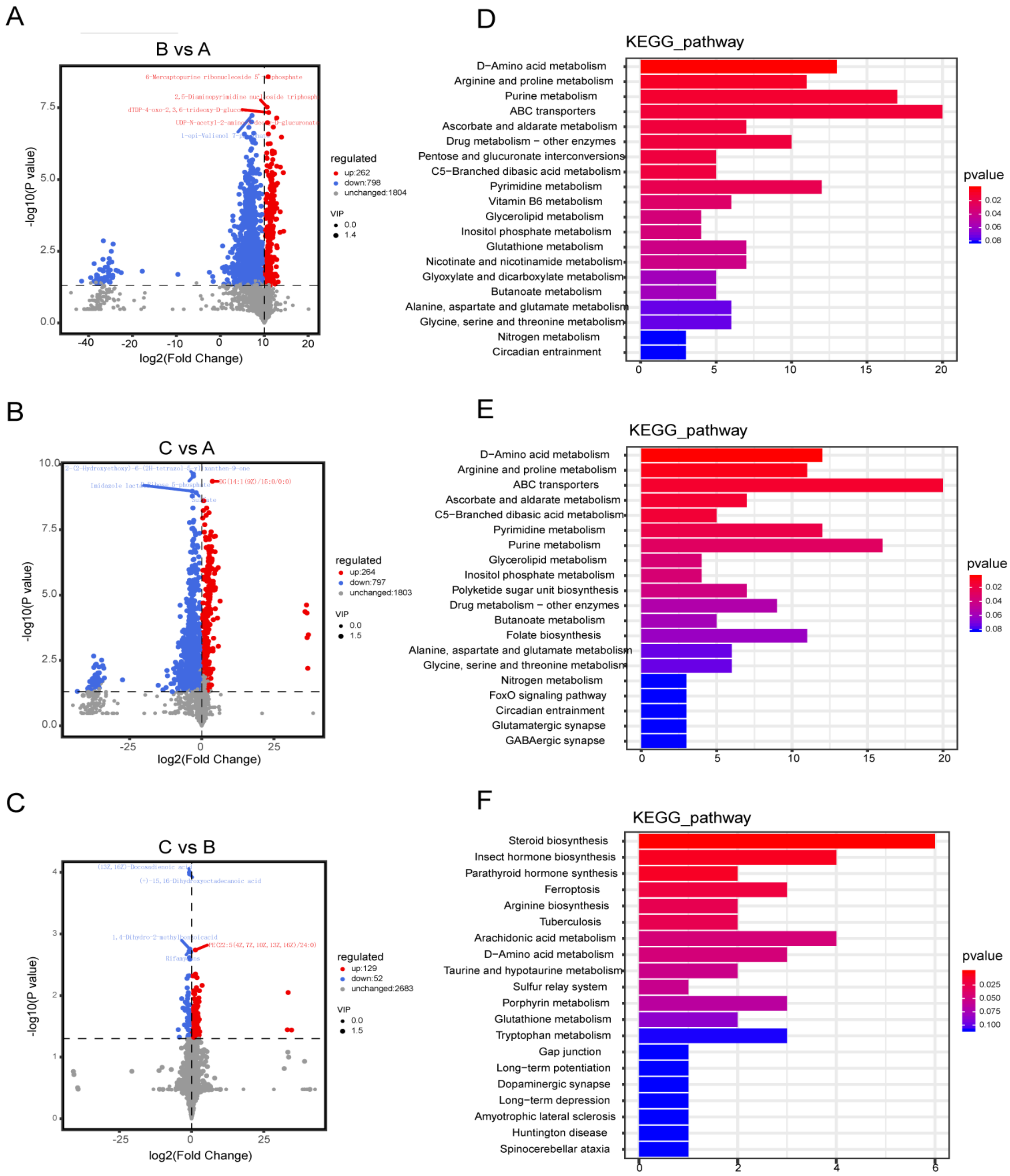

FigureS1 Metabolome analysis. (A) Volcano plots of differential metabolites between A and B groups, (B) C and A, (C) C and B. (D-F) Bar histogram of KEGG enrichment analysis between groups.
